# Indoor Air Pollution Exposure of Women in Adama, Ethiopia, and Assessment of Disease Burden Attributable to Risk Factor

**DOI:** 10.3390/ijerph18189859

**Published:** 2021-09-18

**Authors:** Festina Balidemaj, Christina Isaxon, Asmamaw Abera, Ebba Malmqvist

**Affiliations:** 1 Division of Occupational and Environmental Medicine, Department of Laboratory Medicine, Lund University, 222 42 Lund, Sweden; ebba.malmqvist@med.lu.se; 2 Division of Ergonomics and Aerosol Technology, Department of Design Sciences, Lund University, 223 62 Lund, Sweden; christina.isaxon@design.lth.se; 3 Water and Public Health Department, Ethiopia Institute of Water Resources, Addis Ababa University, Addis Ababa, Ethiopia; asmenst13@gmail.com

**Keywords:** household air pollution, health impact assessment, burden of disease, mortality rates, disease-adjusted life years

## Abstract

Introduction and aim: Air pollution, a major environmental threat to human health, contributes to the premature deaths of millions of people worldwide. Cooking with solid fuels, such as charcoal and wood, in low- and middle-income countries generates very high emissions of particulate matter within and near the household as a result of their inefficient combustion. Women are especially exposed, as they often perform the cooking. The purpose of this study was to assess the burden of disease attributable to household air pollution exposure from cooking among women in Adama, Ethiopia. Methods: AirQ+ software (WHO Regional Office for Europe, Copenhagen, Denmark) was used to assess the health impact of household air pollution by estimating the burden of disease (BoD) including Acute Lower Respiratory Infections (ALRI), Chronic Obstructive Pulmonary Disease (COPD), Ischemic Heart Disease (IHD), lung cancer, and stroke, among a cohort of women in Adama. Household air pollution exposure estimated by cooking fuel type was assessed through questionnaires. Results: Three-quarters (75%) of Adama’s population used solid fuel for cooking; with this, the household air pollution attributable mortality was estimated to be 50% (95% CI: 38–58%) due to ALRI, 50% (95% CI: 35–61%) due to COPD, 50% (95% CI: 27–58%) due to lung cancer, (95% CI: 23–48%) due to IHD, and (95% CI: 23–51%) due to stroke. The corresponding disability-adjusted life years (DALYs) per 100,000 women ranged between 6000 and 9000 per disease. Conclusions: This health impact assessment illustrates that household air pollution due to solid fuel use among women in Adama leads to premature death and a substantial quantity of DALYs. Therefore, decreasing or eliminating solid fuel use for cooking purposes could prevent deaths and improve quality of life.

## 1. Introduction

Air pollution is a major environmental threat to human health, contributing to the premature deaths of millions of people worldwide [1]. A common source of air pollution in low and middle-income countries (LMICs) is cooking with solid fuels, such as charcoal, wood, crop residue, and animal dung [2]. The inefficient combustion of these fuels in traditional household stoves results in very high emissions of particulate matter (PM), carbon monoxide (CO), short-lived climate forcers (such as methane, fluorinated gases, tropospheric ozone, and black carbon) and polycyclic aromatic hydrocarbons (PAHs) [3]. Exposure to these pollutants is pervasive, as an estimated 54% of people living in LMICs rely on solid fuels for cooking [4,5]. When it comes to the disease burden caused by indoor air pollution, its strongest association is observed with lung cancer and COPD. In 2017, indoor air pollution was linked with 1.8 million deaths and 60.9 million DALYs worldwide [6]. While household air pollution has been declining, it still remains as one of the top ten sources of attributable disease burden [7].

Household air pollution is an eminent issue in sub-Saharan Africa as pollutive biomass fuels are frequently used for heating and cooking [8,9,10]. Indeed, the exposed population in this region has increased by 100% since 1980 [5]. In Ethiopia, where 95% of the energy is supplied by biomass sources, indoor air pollution was reported to cause approximately 5% of the national burden of disease and more than 50,000 deaths annually [11]. Indoor concentrations of Addis Ababa are estimated to be 818–905 μg/m^3^ [12,13]. This is about 30 times greater than residential averages in high-income countries [14]. Such exposures tend to disproportionately affect women, whom are most often tasked with cooking, as well as their children [15,16].

Intervention in the continent regarding exposure to indoor air pollution has failed [5]. Due to lack of technical and financial resources, air quality measurements and population exposure assessment are lacking, especially in sub-Saharan Africa [17,18]. This lack of data makes it difficult to develop health statistics and health impact assessments, without which it is almost impossible to develop adequate policies. The lack of measurements and monitoring also reduces public concern and awareness, leading to a lack of policy action and, in turn, lack of encouragement to collect data. Alternatively, greater availability of air pollution statistics can contribute to enhancing public concern, which would in turn lead to primary demand for and the development of clean air strategies [19]. Other benefits of managing air pollution also include the drive for other positive changes, such as in health, well-being, and climate [20].

To address the continued exposure to household air pollution, the World Health Organization (WHO) has developed air quality guidelines describing the great health risks of burning coal, wood, and kerosene indoors [21]. It has also established capacity-building training programs in order to address household air pollution as a risk to human health [22]. While these efforts have filled an important knowledge gap for household energy interventions, more research is needed to understand the health impacts of current indoor energy use and cooking practices among women in sub-Saharan Africa. Additionally, some efforts in reducing air pollution from cookstoves have been relatively successful: China’s National Improved Stove Program, Indian National Program on Improved Chulha, and the Gyapa Stoves Program in Ghana [1]. In the past few years, major advances have also been made in clean fuel available, such as the Indian liquefied petroleum gas program and Ecuador’s electric induction stove program [1]. These initiatives have set a positive example and have enriched knowledge regarding long-term practices of indoor cookstove use in different countries around the world. More detailed evaluations of the health impact of indoor air pollution could potentially support the development of similar programs in Ethiopia.

Health impact assessments (HIA) are decision support tools with the purpose of adding value to the decision-making process by providing an analysis of the potential effects of a project, policy, or program (PPP) on various dimensions of health [23,24,25]. More specifically, HIA seeks to provide decision makers with information to alleviate the negative and maximize the positive impacts on health inequalities and health overall. Ultimately, a successful HIA is one considered by decision makers to inform the development and implementation of a PPP [18,24]. Two groups of factors important for the integration and incorporation of health considerations and HIA findings in decision making include the technical conduct of the HIA and the decision-making environment. Enablers for technical conduct included a consistent methodological approach, timing of the HIA congruent with the decision-making process, inclusion of empirical evidence on health impacts, and shaping recommendations to reflect priorities [23]. Important enablers surrounding the decision-making environment included striking a balance between the HIA credibility and decision maker ownership, policy, statutory and organizational commitment to HIA, and provision of non-controversial and realistic recommendations [26]. Understanding the significance of HIA, the WHO has established a software tool that performs calculations that allow the quantification of health effects of exposure to air pollution in terms of Burden of Disease (BoD), making this methodological approach consistent and credible in assessing health impacts from air pollution exposure and, ultimately, contributing to regional policy change and action [27].

This study aims to enrich the knowledge of health impacts of indoor air pollution exposure from cooking fuels among women in Adama, Ethiopia. Health impact is assessed as burden of disease (BoD), mortality rates, and disease-adjusted life years (DALYs) for Acute Lower Respiratory Infections (ALRI), Chronic Obstructive Pulmonary Disease (COPD), Ischemic Heart Disease (IHD), stroke, and lung cancer.

## 2. Methods

### 2.1. Study Setting and Population

The study setting is the city of Adama, Ethiopia, which has approximately 300,000 inhabitants, a surface area of 29.86 km^2^, a latitude of 8.3229° N, and a longitute of 39.1608° E. Data from the Adama Mother and Child cohort (ClinicalTrials.gov identifier number NCT03305991), a prospective cohort, was used. It consists of women recruited during pregnancy from three public antenatal care clinics in urban Adama (Adama Hospital, Adama Health Centre, and Geda Health Centre). From November 2015 to February 2018, pregnant women were recruited during their first visit to an antenatal care clinic after providing informed consent. The study population included 2085 women, where repeated pregnancies were excluded (*n* = 123). The cohort has previously been used for studying infectious diseases, resulting in three publications [28,29,30].

### 2.2. Exposure Assessment

Questionnaires were used to collect data on indoor air pollution exposure, such as the type of cooking fuel used (charcoal, wood, gas, kerosene, cylinder, or electricity); the frequency of women cooking while pregnant (times per day or times per week); the location of cooking (indoor, outdoor, in the same room as sleeping or separate room, etc.); and the ventilation system, if any, in the living and/or cooking space. In addition, data on education level were obtained and categorized as ‘illiterate’, meaning no formal education was attained by participants; ‘less than 6 years of education’; and ‘6 years or more of education’. At the aerosol laboratory at Lund University, emission factors from different fuels and cooking methods, as well as particle characteristics, are being assessed experimentally. The pregnant women were assumed to be representative for all women in the study area regarding cooking fuel used.

### 2.3. Burden of Disease from Household Air Pollution

**Figure 1:**
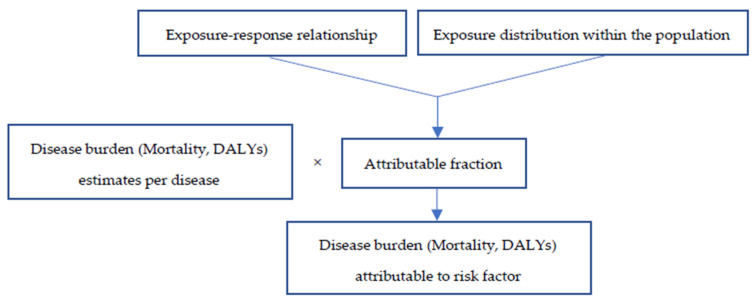
Method utilized for estimating burden of disease attributable to risk factor. DALYs: disease-adjusted life years [27].

In order to quantify burden of disease (BoD) attributable to household air pollution, the population attributable fractions (AF*_p_*) were used by AirQ+ [27]. AF*_p_* represents the proportional devaluation in population mortality and population disease that would arise if the risk factor exposure (in this case, exposure to household air pollution) were to decline to a different optimal exposure scenario (for example, no exposure to household air pollution). The health risks estimates that were used in the AF*_p_* calculations were established by using the methods developed by the Institute for Health Metrics and Evaluation (IHME) [27]. Household air pollution attributable burden was acquired using the AF*_p_*:

$$\mathrm{AF}p=\frac{Pe\left(RR-1\right)}{1+Pe\;\left(RR-1\right)}$$

where *Pe* represents the population percentage exposed to household air pollution by using polluting technologies and fuels for cooking practices and *RR* is the relative risk. Then, AF*_p_s* were applied to each individual disease (ALRI, lung cancer, COPD, stroke, and IHD) in the manner shown in Figure 1 [31,32,33].

The RR values that were used with 95% confidence intervals for each disease analyzed are shown in Table 1. In order to estimate the RR for diseases caused by air pollution exposure, the Global Burden of Disease Study [6,7,27] and WHO [27] developed an Integrated Exposure Response (IER), which was used to estimate and approximate the RR for diseases caused by exposure to air pollution from PM_2.5_ (particulate matter with an aerodynamic diameter < 2.5 µm). The IER merges evidence from epidemiological studies for household air pollution, active smoking, second-hand smoking, and outdoor air pollution in order to estimate the risk of disease (e.g., IHD) at different concentrations of PM_2.5_. This system uses the same mathematical measure or relationship to estimate the risk of e.g., stroke from outdoor air pollution as that of household air pollution or second-hand smoke.

The percentage of the population exposed to PM_2.5_ via household air pollution was attained from the secondary data used in this study, for which the AirQ+ system provided increments of 1 μg/m^3^, and the counterfactual concentration for air pollution was selected between 2.4 and 5.9 μg/m^3^. The exposure–response estimates used for the BoD calculations in AirQ+ software is based on a systematic review by the WHO [27]. The PM_2.5_ exposure level value for women assumed for households relying mainly on polluting technologies and polluting fuels used for cooking was estimated to be 337 μg/m^3^ [32,33].

The country population attributable fractions for stroke, lung cancer, COPD, IHD, and ALRI were calculated using the mathematical formula shown above. The IER function was used to estimate the BoD (mortality and DALYs) due to indoor air pollution of five different causes: ALRI, COPD, IHD, lung cancer, and stroke. For stroke and IHD, there is an age-gradient for the relative risks but presented here are the 95% confidence intervals over their predicted values from the integrated exposure response functions for the age of 70 years [27].

The relative risks for women by cause (Table 1) together with the age-standardized and crude mortality rates as well as estimated DALYs by cause of women of all ages and women aged 15–49 per 100,000 women in Ethiopia (Table 2 and Table 3) were used to estimate the potential effects of indoor air pollution impact in the cohort group.

### 2.4. Statistical Analysis

Frequencies and descriptive statistics of data collected from the questionnaires were assessed using SPSS analysis. The percent of women exposed to pollutants from solid fuel through cooking practices was estimated, which was the only data point from our cohort used directly in the calculations of disease burden. Upon the determination of percentage of exposed population to indoor pollution caused by the use of solid or other fuels for cooking, quantitative research analyses on disease burden attributable to risk factor exposure of pregnant women were assessed. Disease burden studies included acute lower respiratory infections (ALRI), lung cancer, chronic obstructive pulmonary disease (COPD), ischemic heart disease (IHD), and stroke. The studied attributable risk factor was indoor air pollution exposure due to fuel use for cooking purposes.

WHO’s AirQ+ software tool was used for health risk assessment of air pollution. The AirQ+ model is executed using Markov Chain Monte Carlo (MCMC) and similar to other Bayesian analysis, results are a set of full posterior distributions of point estimates of the proportions (of the fuel type usage, by year and by country). As a result, summaries of these distributions can be used to provide means or point estimates as well as uncertainty (e.g., 95% Confidence Intervals) [3].

Results were expressed as BoD attributable to risk factor. Detailed research questions and the analytical approaches of this assessment are shown below.

### 2.5. Research Objectives

The research objectives included the following:

1. Calculating the mortality number of attributable cases using age-standardized versus crude mortality rates per 100,000 women in Ethiopia, for ALRI, COPD, IHD, stroke, and lung cancer;
2. Calculating the mortality number of attributable cases per 100,000 population at risk using age-standardized versus crude mortality rates per 100,000 women in Ethiopia, for ALRI, COPH, IHD, stroke, and lung cancer;
3. Calculating the number of DALYs of attributable cases using all-ages versus ages 15–49 rates per 100,000 women in Ethiopia, for ALRI, COPH, IHD, stroke, and lung cancer;
4. Calculating the number of DALYs of attributable cases per 100,000 women using all-ages versus ages 15–49 rates per 100,000 women in Ethiopia, for ALRI, COPH, IHD, stroke, and lung cancer.

### 2.6. Ethical Considerations

Approval for this study was granted by the Lund University Ethical Committee (Registration numbers: 2015/364 and 2016/576 [amendment] and the Ethical Review Board of the Ministry of Science and Technology, Addis Ababa, Ethiopia (310-046-2015). Written informed consent was obtained from all participants prior to any study procedures. Data were collected and processed strictly under code and stored in a REDCap (www.project-redcap.org; accessed on 1 August 2021) database hosted by the Lund University Faculty of Medicine in Sweden. This was done in order to preserve study participant confidentiality and integrity.

## 3. Results

### 3.1. Characteristics of the Population Sample

The final population sample was composed of 2084 participants, between ages 13 and 40, living in Adama, Ethiopia and its surroundings. The majority of the cohort was between the ages of 18 and 29 (83%), and most were married (96%). The leading occupation among the cohort group was homemaker (63%), and 56% of the study population had between six and 12 years of education.

Regarding the use of fuel type, 75.4% reported using solid fuels (75% used charcoal or wood, and 0.4% used animal dung), 48% used electricity, 4.0% used gas or kerosene, and 0.8% used a liquid petroleum gas (LPG) cylinder. Out of those reported using electricity for cooking, around half used it in combination with solid fuels, leaving 23% using solely electricity. In addition, 96% of the cohort group reported having electricity at home. Smoking percentages were low: 0.1% for current smokers, 0.2% for previous smokers that stopped for this pregnancy, and 1.2% for smokers in household.

### 3.2. Burden of Disease (BoD) Assessments Attributable to Risk Factor, AirQ+

The household air pollution attributable mortality is just over 49.5% (95% CI: 37.6–57.6%) due to ALRI, 49.5% (95% CI: 34.5–61.3%) due to COPD, (95% CI: 23.2–47.5%) due to IHD, 49.5% (95% CI: 27.4–57.6%) due to lung cancer, and (95% CI: 23.2–51.4%) due to stroke.

Disease burden mortality estimations of attributable cases for the study representative population using age-standardized mortality rate per 100,000 women in Ethiopia are 84 (95% CI: 64–98) for ALRI, 22 (95% CI: 15–27) for COPD, (95% CI: 53–106) for IHD, 38 (95% CI: 21–44) for lung cancer, and (95% CI: 40–88) for stroke. The burden of disease mortality estimations and the mortality number of attributable cases for the study representative population using a crude mortality rate per 100,000 women in Ethiopia give a lower estimated number of cases (see Table 4).

Disease burden DALYs estimations of attributable cases for the study representative population using DALYs per 100,000 women of all ages in Ethiopia are 20,566 (95% CI: 15,632–23,922) for ALRI, 15,541 (95% CI: 10,846–19,243) for COPD, (95% CI: 25,316–51,897) for IHD, 13,923 (95% CI: 7701–16,195) for lung cancer and (95% CI: 23,308–51,656) for stroke. These estimated attributable cases are lower if only women aged 49 and under are considered (Table 5).

## 4. Discussion

### 4.1. Implications of the Study

This study adds to the existing, but limited, literature on the health effects of indoor air pollution on women. Even though 96% of the Adama population has electricity at home, 75.4% use solid fuel for cooking. As a result, the estimated household air pollution attributable mortality is 49.5% (95% CI: 37.6–57.6%) due to ALRI, 49.5% (95% CI: 34.5–61.3%) due to COPD, (95% CI: 23.2–47.5%) due to IHD, 49.5% (95% CI: 27.4–57.6%) due to lung cancer, and (95% CI: 23.2–51.4%) due to stroke. The corresponding disability-adjusted life years (DALYs) per 100,000 women were in the order of 6000–9000 per disease. Previous studies of household air pollution in Ethiopia have found rates of using firewood in rural areas to be around 90% and 54% in urban areas. Charcoal was almost never used in rural areas but stood for 18% of fuel used in urban areas [34,35]. Therefore, the potential health impact of switching to cleaner cooking fuels can be substantial. Similar methods have previously been used to assess the health burden of ambient air pollution in Addis Ababa, which found that over 2000 premature deaths could have been avoided if air pollution levels were reduced to WHO air quality guidelines [36].

While previous research [1,7] has shown the detrimental effects that particulate matter has on the human body, this study instead focused on pregnant women and analyzed the health impact of their daily choices regarding solid fuel use for cooking. In estimating the burden of disease attributed specifically to cooking practices, awareness is raised, and this knowledge, in turn, can lead to potential interventions to reduce exposure or even avoid it altogether.

As previously mentioned, 96% of the cohort reported having electricity at home, yet only 48% reported using electricity for cooking, and this was often in combination with other fuels. Despite its availability, the reliability of electricity cannot be assumed, as power shortage is common in Adama. While using electricity is undeniably better for one’s health than burning solid fuels, there are many factors that can profoundly influence the choice of cooking fuel within a household even when electricity is available. Leading factors include socioeconomic factors (household size and income; household head’s education level, gender, and age; and location of household), technological changes for sources of energy, cultural preferences of energy choice, and shifts in energy supply options and prices [37,38,39]. In addition, cooking with solid fuels is entwined with structural and organizational elements, such as practices that are established traditions, create a sense of belonging, follow gender norms, and generate income [40]. People living in poverty are also likely to be consumed with immediate needs and circumstances, worries about managing unreliable, and small cash flows earned through uncertain jobs. As a result, they are less likely to prioritize future needs, including their long-term health. This phenomenon of poverty using up cognitive capacity has been termed the “bandwidth tax” [41] and has not been considered in any study on the barriers of adoption of clean energy [41]. Behavior change is already difficult to achieve, but it becomes especially hard for families living in poverty [41].

Cookstove intervention studies in Ghana have found significantly lower air pollution levels when solid fuels were replaced with liquefied petroleum gas [42]. However, such an initiative would still require reliable, affordable, and subsidized access and delivery [41]. In addition, providing education on health effects of solid fuel and possible strategies on fuel use such as cooking outdoors instead of indoors may be simple and feasible steps that can be taken by women to reduce their exposure. In this context, it might also be beneficial to compare indoor air pollution exposure to known detrimental effects from passive smoking as was done by Ambade et al. [43].

### 4.2. Policy Implications

In order to coordinate health systems and the populations they assist, policymakers need to first be able to understand the true nature of the health challenges that their countries face as well as how those health challenges are shifting over time. This means that besides disease prevalence estimates, more knowledge, such as the number of people affected by disease, is required. Burden of disease estimations incorporate not only knowledge regarding disease prevalence and risk factors but also an understanding of the relative harm that the disease causes in terms of quality-adjusted life years and mortality rates. Subsequently, this information can provide decision makers with the ability to compare, for example, the societal and financial costs of various diseases and allow them to directly apply it. Indeed, the output of such burden of disease estimations are described in terms of the number of deaths per 100,000 population or DALYs per 1000 population, which is a comprehensible formulation, regardless of policy and decision makers’ backgrounds.

The results of this study may serve not only as basis for health systems policy implications but also for environmental policy considerations and development. However, the regulation and governance of air pollution have been shown to be a considerable challenge in sub-Saharan African cities. While regional and global initiatives for monitoring and regulation systems exist, they are rarely prioritized within the political agenda. Regulatory frameworks are mostly nonexistent or weak, and most countries undergo poor knowledge transfer regarding health effects and health risks of air pollution [44]. While a few countries in Africa have incorporated the WHO’s air quality guidelines into their national legislation, enforcing and monitoring laws tend to be beyond the current capacity of many governments. In addition, the African legislation of air pollution focuses mostly on industries, while the most prominent sources of pollution include domestic biomass burning and transportation [45,46,47].

### 4.3. Merits and Limitations of the Study

This study is among the few studies that assess household air pollution exposure due to solid fuel use in sub-Saharan Africa. It also serves as the first study of its type to take place in Adama, Ethiopia, a city in Ethiopia having a high percentage of the population with electricity available in the homes (94.8%) when compared to rural areas. The World Bank has previously estimated that only 30% of the population has access to electricity [48]. The merits also include a well-established method for estimating health impacts and data from a questionnaire among more than 2000 women in a peri-urban area [49]. Additionally, the rate of smoking, an uncommon practice among women in Ethiopia, was low in our cohort, as mentioned previously: 0.1% for current smokers, 0.2% for previous smokers that stopped for this pregnancy, and 1.2% for smokers in household. Therefore, the assumption to only consider cooking exposure was appropriate for this study population and did not likely contribute to exposure misclassification.

Exposure to household air pollution in this study only considers exposure generated by the use of cooking fuel(s). Heating and lighting fuels have not been included, both of which may increase the overall exposure of study participants to household air pollution. However, our study setting is located in a climate where heating is not required. Ambient air pollution can also penetrate the house, but this effect is often calculated separately, which has been done by Abera et al. through measurements of NO_x_ in Adama [50]. In addition, the IER function assumes the toxicity of PM_2.5_ from household air pollution and ambient air pollution to be approximately the same, which is likely not the case, as their sources is unique. Additionally, the age-standardized and crude mortality rates and estimated DALYs for women of all ages and women of ages 15–49 for lung cancer in Ethiopia were not available from WHO data. Instead, these had to be adopted from other sources representing global and United States lung cancer mortality rates and DALYs, respectively, obtained from Research Gate and USstatscenter.gov. Therefore, the results estimated for lung cancer are not representative per 100,000 women in Ethiopia, specifically, but for women globally (mortality rates) and women in the U.S. (DALYs). Another possible limitation could be that we assume that cooking fuels are the same for pregnant and not pregnant women. As pregnancy rates are high in Ethiopia and fuel changes are slow, we believe this is a fair assumption. Finally, this study cannot claim to be representative of rural areas of Ethiopia or of the entire country, as the availability of electricity and cooking fuel use may vary.

### 4.4. Recommendations for Future Research

While charcoal is widely used for heating, lighting, and cooking purposes, this study only assessed use of charcoal for cooking purposes. In order to reach a more accurate estimate of the percentage of population using solid fuel and being exposed to its pollutants, future studies should assess the use of coal for heating in addition to cooking. While heating is not needed in Adama, other parts of Ethiopia and other countries in Sub-Saharan Africa do require heating.

This study included a cohort of pregnant women, 75.4% of whom used solid fuels for cooking purposes and 72.9% of whom used them twice a day, leading to the knowledge that their unborn children were exposed to hazardous concentrations of household air pollutants while still in the womb. Future studies in Adama and similar settings should strongly consider research on the impacts of household air pollution on pregnancy-related diseases, adverse birth outcomes, and the health effects among children who were exposed in utero. However, there are no risk estimates for these impacts included in the WHO’s AirQ+ as of today.

Lastly, there is currently a lack of data regarding lung cancer mortality rates and DALYs for Ethiopia by the WHO’s Department of Information, Evidence, and Research. The closest disease covered through these data is mesothelioma. Considering the detrimental respiratory and systemic health effects that pollution has, it is recommended that this database is updated with lung cancer metrics for both men and women residing in sub-Saharan Africa.

## 5. Conclusions

This study enriches the knowledge of the health impacts of household air pollution exposure on women through burden of disease assessments, including mortality rates and DALYs for acute lower respiratory infections, lung cancer, chronic obstructive pulmonary disease, ischemic heart disease, and stroke. The results can contribute to increasing the principal knowledge, which is necessary for suitable policymaking.

## Figures and Tables

**Table 1:** Relative risks (RR) for ALRI, COPD, IHD, lung cancer, and stroke, for women, caused by exposure to air pollution from PM_2.5_. Please note that the central values are not provided for IHD and stroke but only the 95% Confidence Interval (CI) of their predicted values.

Disease	RR (95% CI)	Reference
Acute lower respiratory infection	2.3 (1.8–2.8)	[6,7,27]
Chronic obstructive pulmonary disease	2.3 (1.7–3.1)	[6,7,27]
Ischemic heart disease	(1.4–2.2)	[6,7,27]
Lung cancer	2.3 (1.5–2.8)	[6,7,27]
Stroke	(1.4–2.4)	[6,7,27]

**Table 2:** Age-standardized and crude mortality rates per 100,000 population of women in Ethiopia, Africa.

Disease	Age-Standardized Mortality Rates	Crude Mortality Rates
Acute lower respiratory infection	75.4	57.8
Chronic obstructive pulmonary disease	19.7	8.7
Ischemic heart disease	98.9	42.2
Lung cancer	33.6	31.9
Stroke	75.6	33.9

Data obtained from the World Health Organization Department of Information, Evidence and Research. Data originally collected in 2016, published in April 2018, accessed in April 2020; the WHO’s International Agency for Research on Cancer Globocan, 2018. Lung cancer rates obtained from USstatscancer.gov.

**Table 3:** Estimated DALYs by cause per 100,000 population of women of all ages and of ages 15–49 in Ethiopia, Africa.

Disease	Disease-Adjusted Life Years: All Ages	Disease-Adjusted Life Years: Ages 15–49
Acute lower respiratory infection	18,368	9825
Chronic obstructive pulmonary disease	13,880	1450
Ischemic heart disease	48,300	4930
Lung cancer	12,435	141
Stroke	44,470	5010

Data obtained from the World Health Organization Department of Information, Evidence and Research. Data originally collected in 2016, published in April 2018, and accessed in April 2020; the WHO’s International Agency for Research on Cancer Globocan, 2018 and Research Gate, 2018.

**Table 4:** Burden of disease (BoD) estimations by AirQ+ as total mortality number of attributable cases and mortality number of attributable cases per 100,000 population at risk, using age-standardized and crude mortality rates per 100,000 women in Ethiopia, Africa.

Mortality Rate Type	Burden of Disease	ALRI	COPD	IHD	Lung Cancer	Stroke
Utilizing age-standardized mortality rates per 100,000 women in Ethiopia	Estimated Attributable Proportion (%)	49.5 (37.6–57.6)	49.5 (34.5–61.3)	(23.2–47.5)	49.5 (27.4–57.6)	(23.2–51.4)
	Number of Attributable Cases	84 (64–98)	22 (15–27)	(52–106)	38 (21–44)	(40–88)
	Number of Attributable Cases per 100,000 Population at Risk	37.3 (28.4–43.4)	9.8 (6.8–12.1)	(22.9–47.0)	16.6 (9.2–19.4)	(17.5–38.8)
Utilizing crude mortality rates per 100,000 women in Ethiopia	Number of Attributable Cases	65 (49–75)	10 (7–12)	(22–45)	36 (20–42)	(18–39)
	Number of Attributable Cases per 100,000 Population at Risk	28.6 (21.8–33.3)	4.3 (3.0–5.3)	(9.8–20.1)	15.8 (8.7–18.4)	(7.9–17.4)

**Table 5:** Burden of disease (BoD) estimations by AirQ+ as total number of DALYs of attributable cases and number of DALYs of attributable cases per 100,000 population at risk, using DALYs of all ages and DALYs of ages 15–49 per 100,000 women of all ages in Ethiopia, Africa.

Mortality Rate Type	Burden of Disease	ALRI	COPD	IHD	Lung Cancer	Stroke
Utilizing disease-adjusted life years for all ages of women in Ethiopia	Estimated Attributable Proportion (%)	49.5 (37.6–57.6)	49.5 (34.5–61.3)	(23.2–47.5)	49.5 (27.4–57.6)	(23.2–51.4)
	Number of Attributable DALYs	20,566 (15,632–23,922)	15,541 (10,846– 19,243)	(25,316–51,897)	13,923 (7701–16,195)	(23,308–51,656)
	Number of Attributable DALYs per 100,000 Population at Risk	9092.2 (6910.9–10,575.7)	6870.6 (4795.0–8507.2)	(11,191.8–22,943.0)	6155.3 (3404.5–7159.7)	(10,304.4–22,836.4)
Utilizing disease-adjusted life years for ages 15–49 for women in Ethiopia	Number of Attributable DALYs	11,001 (8362–12,796)	1624 (1133–2010)	(2584–5297)	158 (87–184)	(2626–5820)
	Number of Attributable DALYs per 100,000 Population at Risk	4863.4 (3696.6–5656.9)	717.8 (500.9–888.7)	(1142.4–2341.8)	69.8 (38.6–81.2)	(1160.9–2572.8)

## Data Availability

Due to ethical reasons and limitations set by The Ethical Boards personal data can’t be shared.

## Abbreviations

AF*_p_*	Attributable Fraction of the Population
ANC	Antenatal Care
ALRI	Acute Lower Respiratory Infection
ASDR	Age Standardized Death Rate
BoD	Burden of Disease
CDC	Center for Disease Control
CFCs	Chlorofluorocarbons
CI	Confidence Interval
COPD	Chronic Obstructive Pulmonary Disease
DALYs	Disease-Adjusted Life Years
DDT	Dichlorodiphenyltrichloroethane
GBD	Global Burden of Disease
IER	Integrated Exposure Response Function
IHD	Ischemic Heart Disease
LMIC	Low-Income and Middle-Income Countries
MCMC	Markov chain Monte Carlo
PM_2.5_ & PM_10_	Particulate Matter 2.5 μm and Particulate Matter 10 μm
PAF	Population Attributable Fraction
PAHs	Polycyclic Aromatic Hydrocarbons
*Pe*	Percentage of Population Exposed to Household Air Pollution
PCBs	Polychlorinated Biphenyls
RR	Relative Risk
SPSS	Statistical Package for Social Sciences
USEPA	United States Environmental Protection Agency
WHO	World Health Organization
PPP	Project, Policy, Program
